# International Benchmarking of Pharmacology Curricula and Prescribing Related Learning Outcomes, Implications for Australian Health Professional Education: A Systematic Review and Meta-Analysis

**DOI:** 10.3390/pharmacy14010027

**Published:** 2026-02-03

**Authors:** Syed Haris Omar, Anna Barwick

**Affiliations:** 1 School of Dentistry and Medical Sciences, Faculty of Science and Health, Charles Sturt University, Orange, NSW 2800, Australia; 2 School of Rural Medicine, University of New England, Armidale, NSW 2351, Australia

**Keywords:** pharmacology education, integrated curriculum, prescribing competency, medical education, global health education, curriculum benchmarking

## Abstract

Background: Pharmacology plays a central role in linking biomedical science concepts with their application in clinical practice across medical and healthcare education. Globally, the pharmacological curriculum has evolved, just like other disciplines, through the integration of case-based, problem-based, and hybrid teaching models that led to firm clinical reasoning and long-term learning. Thus, this study aims to evaluate and compare the learning outcomes of pharmacology curricula across the globe by adopting a systematic review and meta-analysis research approach. Methods: This comprehensive review was conducted with transparency and integrity in accordance with Preferred Reporting Items for Systematic Reviews and Meta-Analyses (PRISMA 2020) guidelines and was registered with PROSPERO (CRD420251207753). Five electronic databases, including MEDLINE (PubMed), EMBASE, CINAHL, PsycINFO, and the Cochrane Library were searched from January 2000 to October 2025. The Cochrane Library tool was used for the risk of bias assessment of randomised controlled trials, while the Joanna Briggs Institute (JBI) checklist was used for mixed-design, quasi-experimental, and cross-sectional cohorts. Review Manager 5.4 was used for statistical analysis. Results: Out of 3300 identified studies, 11 met the inclusion criteria, spanning 11 countries (published between 2007 and 2025). Integrated and case-based curricula significantly improved pharmacology knowledge compared to traditional lecture-based methods (SMD = 0.35; 95% CI: 0.07–0.64; *I*^2^ = 75%). Student satisfaction also favours integrated learning (OR = 1.53; 95% CI: 1.16–2.02; *I*^2^ = 46%). Most included studies were of moderate-to-high methodological quality. Conclusion: Globally, active and integrated pharmacology curricula foster greater cognitive understanding and learner satisfaction than conventional models. However, significant variability persists in resource-limited settings, leading to unequal competency in prescribing and therapeutic reasoning. Australian pharmacology programmes align broadly with international standards but require greater standardisation in assessment and experiential learning.

## 1. Introduction

Pharmacology is a core discipline that supports the integration of biomedical science knowledge with clinical decision-making in medical and healthcare education. This subject provides basic knowledge of understanding drug mechanisms, therapeutic applications, and safety outcomes of drugs to future healthcare professionals [1]. With the discovery of novel drug classes, complex treatment protocols, and individualised medicines, the pharmacology curriculum prepares graduates for safe therapeutic decision making as part of a broader clinical and professional education framework [2]. Despite its growing significance, the pharmacological curriculum has been facing wide disparities in various global regions, such as differences in methods of teaching, as well as assessment that result in variations in learning outcomes and students’ competencies [3].

Over time, the curriculum of pharmacological education has evolved just like other disciplines by the integration of case-based, problem-based, and hybrid teaching models that led to firm clinical reasoning and long-term learning [4]. The shifting of discipline-based learning to an integrated curriculum has aimed to improve students’ knowledge about pharmacological concepts in clinical settings [5,6]. Various frameworks exist, including competency-based medical education (CBME), wherein curriculum content is produced by determining the tasks or abilities that students must acquire for specific developmental outcomes [7]. Furthermore, in outcome-based education (OBE), the curriculum is developed according to specified educational outcomes and anticipated student learning results [8]. However, the ways of adopting these integrated approaches differ significantly across countries or regions due to variations in resource availability, educational systems, and cultural perceptions.

International diversity in pharmacology education is extensively affected by national educational policies, socioeconomic factors, and institutional priorities. In high-income countries, pharmacology curricula have been incorporated with digital simulations, multidisciplinary learning, pharmacovigilance exercises, and student-focused pedagogies to enhance students’ knowledge [9]. However, many low- and middle-income countries (LMICs) utilise traditional lecture-based learning due to resource limitations, limited infrastructure, and faculty shortages [10,11]. These differences indirectly shape the clinical competencies of graduates (e.g., their readiness for management of complex pharmacotherapeutic scenarios).

**Figure 1:**
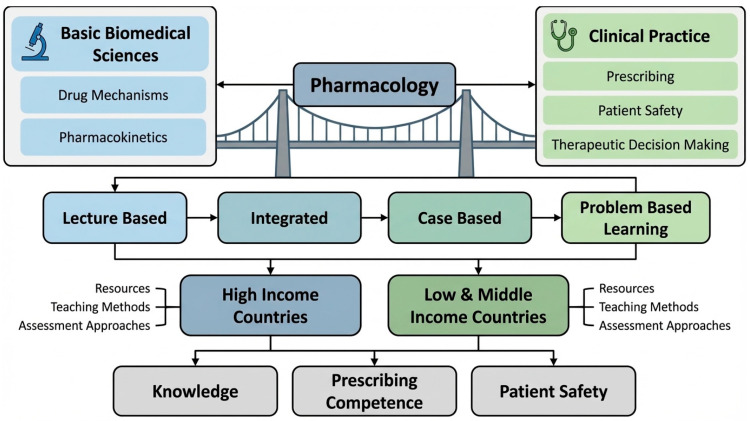
Conceptual framework illustrating pharmacology as a bridge between basic biomedical sciences and clinical practice, highlighting international variation in teaching models and their impact on prescribing competence and patient safety. The figure was created by the authors using standard diagramming software.

On the other hand, learning outcomes serve as a major indicator to understand or evaluate the effectiveness of any curriculum, including pharmacological education. These indicators measure the cognitive knowledge, practical skills, prescribing behaviours, and ethical responsibility in therapeutic management (Figure 1) [12]. Modern assessment tools, such as objective structured clinical examinations (OSCEs), feedback-based surveys, and self-assessment questionnaires, have made the evaluation of learning outcomes of pharmacological curricula feasible [13]. However, due to variations in educational evaluation instruments and contextual factors, including student demographics, healthcare system demands, and cultural views on drug use, comparing these results across curricula is difficult. Some researchers have tried to measure knowledge competencies through objective structured clinical examinations (OSCEs), prescription safety checks, or pre- and post-intervention assessments [14,15]. However, few studies have systematically combined these findings to find out which pedagogical strategies effectively enhance pharmaceutical competence or to identify international trends [16,17].

Evidence suggests that a number of key curriculum features are linked to an improvement in readiness to work with drugs pharmacologically; these include vertically integrating pharmacology with clinical content, using both case based and problem-based learning to provide context for drug therapy, aligning assessment with actual prescribing tasks (e.g., calculating doses, identifying adverse drug reactions), and structured feedback provided through OSCEs and simulations (to emphasise application and clinical reasoning over recalling facts).

The globalisation of healthcare education has highlighted the need for comparing various pharmacological curricula. As healthcare professionals move across borders, there is an immense need for uniformity in pharmacy educational standards. Various organisations such as the World Health Organisation (WHO) [18], World Federation for Medical Education (WFME) [19] and the International Association of Medical Science Educators (IAMSE) [20] have emphasised the implications of a uniform framework in medical education globally. Even with strict implications of medical education standards across the globe, local adaptations of pharmacology curricula remained a challenge. Some countries have successfully implemented integrated curricula, while others relied on understanding the basic pharmacological principles in their curricula. Thus, disparities in students’ preparedness for effective prescribing become a significant concern in global healthcare settings [4,21].

Australian pharmacology and medical programmes are globally recognised for their strong focus on evidence-based medicine, clinical pharmacology, and patient safety. However, disparities exist across Australian universities, such as course designs, varied experiential learning, and credit allocations [22]. The Pharmaceutical Society of Australia’s Competency Standards [23] and the Australian Medical Council’s accreditation frameworks [24] have been working to align the pharmacology training with international standards of clinical practice. Generally, pharmacology is a compulsory subject for undergraduate programmes of medicine, pharmacy, nursing, and dentistry schools (Figure 1). However, differences exist in curriculum designs, assessment tools, and integration with clinical subjects. While some colleges continue to use traditional lecture-based training, others have adopted case-based and interprofessional teaching methodologies. These discrepancies could affect how prepared students are to prescribe safely after graduation [25]. Although Australian graduates typically exhibit high pharmacological knowledge, reports from the Australian Medicines Handbook and medical licensing exams indicate that areas like dose calculation, adverse drug response detection, and polypharmacy management continue to be problematic [26]. To find both strengths and areas for growth, Australian curricula must be compared to international standards. Additionally, questions remain regarding how Australian pharmacology programmes compare globally in terms of curricular structure, teaching strategies, and graduates’ preparedness for safe prescribing.

Numerous studies have reviewed the pharmacology education in individual countries and institutions to analyse their pedagogical approaches [9,27]. But few studies have analysed the learning outcomes of pharmacological curricula across the regions to identify global patterns [28]. Previous studies have also reported the challenges, such as a lack of standardisation in assessments, information overload, insufficient clinical integration, and disconnection between pharmacology teaching and real-world prescribing practices [29]. These challenges are common in LMICs, where curricula do not meet modern global educational standards [20]. Contrarily, institutions of developed countries have other types of issues, such as a lack of student engagement and clinical relevance of basic pharmacological concepts.

Additionally, there are implications for healthcare delivery from comprehending worldwide trends in pharmacology education. Safe and efficient prescription practices, medication adherence, and patient education, all essential elements of high-quality healthcare systems, are supported by pharmacology [30]. Public health concerns arise from prescription errors, adverse drug responses, and irrational drug usage, all of which are caused by pharmacological knowledge gaps [31]. Therefore, patient safety and the effectiveness of the health system depend on making sure that healthcare graduates generally have the necessary pharmacological competence. Thus, a globally informed assessment of pharmacology courses can help address one of the underlying reasons for variation in treatment results and prescribing patterns. Keeping these considerations in view, this systematic review and meta-analysis aims to compare pharmacology curricula and associated learning outcomes across different global regions, especially Australia.

## 2. Objectives

- Map pharmacology curricula designs worldwide and in Australia.
- Compare learning outcomes and prescribing competence across countries.
- Quantify effects of teaching approaches on knowledge and prescribing outcomes.
- Identify gaps and priorities for Australian programmes.

## 3. Key Questions

- How do Australian curricula differ from international models in content, integration, and assessment?
- Which approaches improve pharmacology knowledge and prescribing outcomes?
- What is Australia’s relative position on competency frameworks and core concepts adoption?

## 4. Methods

This systematic review and meta-analysis were undertaken to evaluate and compare the learning outcomes of various pharmacology Curricula across the globe. This comprehensive review was conducted with transparency and integrity in accordance with Preferred Reporting Items for Systematic Reviews and Meta-Analyses (PRISMA 2020) guidelines [32]. The review protocol was prospectively registered in the International Prospective Register of Systematic Reviews (PROSPERO; registration number CRD420251207753). The PRISMA 2020 checklist is provided in the Supplementary Materials.

### 4.1. PICOS Framework

This study has used the Population Intervention Control Outcome Study design (PICOS) framework to guide the search as shown in Table 1 [33].

The PICOS Framework was used to select studies and synthesise data in this study. The Population element identified that undergraduate and postgraduate health professional students were included in the study. The intervention element included any structured pharmacology or clinical pharmacology educational approach beyond traditional lecture-based education. The comparator element identified conventional or lecture-based educational approaches. The outcomes element included only measurable indicators of knowledge, competence, or perceptions of learners. The Study Design Criteria element provided a way to ensure inclusion of empirical studies with extractable data to allow for qualitative or quantitative analysis.

### 4.2. Eligibility Criteria

#### 4.2.1. Inclusion Criteria

Studies were included if they evaluated or described pharmacology curricula in undergraduate or postgraduate programmes in medicine, pharmacy, dentistry, or nursing. Eligible studies compared curriculum designs, teaching approaches, or learning outcomes across institutions, regions, or countries. Both quantitative and qualitative studies were considered, including cross-sectional surveys, cohort studies, randomised trials, and mixed-methods research assessing educational interventions or prescribing competence. Studies that reported measurable outcomes such as student knowledge, prescribing skills, learning satisfaction, or assessment performance were included. Publications focusing on curriculum mapping, integration of pharmacology teaching, or innovations such as problem-based learning, simulation, or digital education were also eligible. Peer-reviewed journal articles published in English, regardless of publication year, were included. Reports providing relevant data on the Australian pharmacology curriculum in comparison to international programmes were prioritised to facilitate benchmarking and global curriculum mapping.

#### 4.2.2. Exclusion Criteria

Studies were excluded if they did not focus on pharmacology education or if pharmacology was only a minor or unspecified component of a broader medical or health sciences curriculum. Articles that did not evaluate or compare curricular structure, teaching methodologies, or learning outcomes were omitted. Editorials, commentaries, opinion pieces, conference abstracts, book chapters, and review papers without original data were excluded. Studies limited to continuing professional development, postgraduate specialisation (e.g., clinical pharmacology fellowships), or non-degree courses were not considered. Excluded from the studies were those that did not explicitly state how pharmacology was integrated into the curriculum or as an educational intervention; did not report defined learning outcomes (knowledge, competence, or perceptions); or did not provide transparent descriptions of their methods or samples; or could not be extracted for analysis.

Non-English publications without available English translations were also excluded. Duplicate publications, preprints without peer review, and studies with overlapping data sets from the same cohort were removed to ensure the integrity and originality of included evidence.

### 4.3. Search Strategy

A complete investigation of the literature was done to find authentic studies or studies matching PICO. A systematic search was conducted across five electronic databases, including MEDLINE (PubMed), EMBASE, CINAHL, PsycINFO, and the Cochrane Library, which were searched from January 2000 to October 2025. The search strategy for PubMed was (“Pharmacology”[Mesh] OR pharmacology education OR pharmacology curriculum OR pharmacology teaching OR pharmacology training) AND (curriculum OR course content OR syllabus OR learning outcomes OR educational objectives) AND (global OR international OR cross-country OR worldwide OR regional comparison OR multinational) AND (“Students, Pharmacy”[Mesh] OR “Students, Medical”[Mesh] OR health sciences students OR pharmacy students OR medical students) and similar was used for other databases. All previously published meta-analyses and systematic reviews on similar topics were searched to reach authentic data.

### 4.4. Study Selection & Data Extraction

The titles and abstracts of included studies were separately examined by two reviewers to determine whether they met the inclusion criteria. For ultimate inclusion, full-text publications of potentially qualifying research were collected and evaluated. Any conflicts or differences between reviewers were settled by discussion or, if required, by consulting a third reviewer. The following information was extracted from each included study: study characteristics (authors, year of publication, study design), study population or settings, type of curriculum, comparator details, outcome measures, and findings.

### 4.5. Methodological Quality Assessment

The Joanna Briggs Institute (JBI) critical appraisal checklist used methodological quality assessment of included cohort studies, mixed methods, and quasi-experimental studies for this meta-analysis. The methodological quality of the included cohort studies or empirical studies and the strategies they employed to address and minimise bias were evaluated using the JBI critical assessment instrument. Based on the methodology of studies, there are JBI-standardised appraisal instruments suitable for JBI reviews of efficacy [34].

### 4.6. Risk of Bias Assessment

The Cochrane risk of bias tool was applied to assess the risk of bias of included RCT’s. The risk bias of included studies was evaluated on the basis of six domains; allocation concealment, blinding of participants, selection bias, blinding of outcome assessment, selective reporting, and other bias. The score or level of each included study was categorised into low risk, unclear, and high risk [35].

### 4.7. Statistical Analysis

Any disagreement was resolved by discussion with a senior reviewer to reach a consensus. The Review Manager 5.4 statistical software (The Cochrane Collaboration, Copenhagen, Denmark) was used for this systematic review and meta-analysis [36]. Dichotomous variables were analysed through odds ratios (OR) and 95% confidence intervals (CI), while continuous variables were analysed by standardised mean differences (SMD) and 95% CI. We used Hedges g for continuous outcomes with means and standard deviation (SD) [37]. Chi-squared tests, *I*^2^ statistics, and visually represented forest plots were used to evaluate statistical heterogeneity. When analysing data with *p* ≤ 0.05 and *I*^2^ ≥ 50%, the random effects model was employed. The data with *p* > 0.05 and *I*^2^ < 50% were analysed using the fixed effect model. The results of the analysis were deemed statistically significant when the two-sided *p*-values were less than 0.05.

## 5. Results

### 5.1. Study Selection

**Figure 2:**
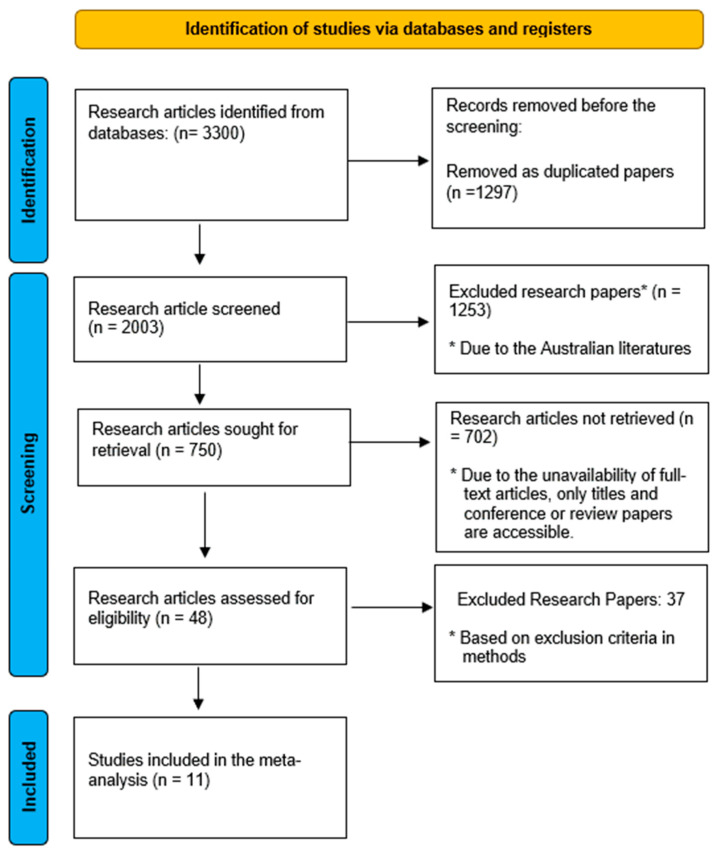
PRISMA Flow chart for screening and selection of included studies. Asterisk highlighted the reason/explanation for task done.

In this systematic review and meta-analysis, the PRISMA guidelines were followed in the selection and screening of research articles pertaining to the study aim “International Comparison of Pharmacology Curricula and Learning Outcomes.” Database searches produced a total of 3300 items, of which 2003 remained after duplicates and insufficient text were eliminated (Figure 2). After an initial screening of 2003 research publications, 750 papers were searched for retrieval. There were only 48 publications that met the eligibility requirements, and there were ultimately 36 research articles excluded, with 11 included in the analysis. The primary reason studies were excluded at the full-text level was a lack of measurable learning outcomes, pharmacology as part of the curriculum but as a secondary or unspecified component, too little detail regarding methodology, or duplicate data for the same group.

### 5.2. Characteristics of Included Studies

A total of eleven studies were published between 2007 and 2025, reflecting the earliest eligible study identified after screening across diverse regions, including Saudi Arabia, the United Kingdom, Israel, Iran, Turkey, Ethiopia, China, India, Pakistan, and the Netherlands, were included in this systematic review (Table 2). Study designs encompassed randomised controlled trials, quasi-experimental, cohort, cross-sectional, and mixed-methods approaches, with sample sizes ranging from 76 to 1275 students. Most studies evaluated medical or health science undergraduates using lecture-based, integrated, blended, or active learning models. Assessment tools commonly included electronic surveys, final examinations, and validated Likert-scale questionnaires to measure pharmacology knowledge and student satisfaction. Institutions such as King Saud bin Abdul-Aziz University (Saudi Arabia), St. George’s University (UK), Tehran University (Iran), and Utrecht University (The Netherlands) represented international diversity in curriculum structures.

### 5.3. Risk Bias Assessment

**Figure 3:**
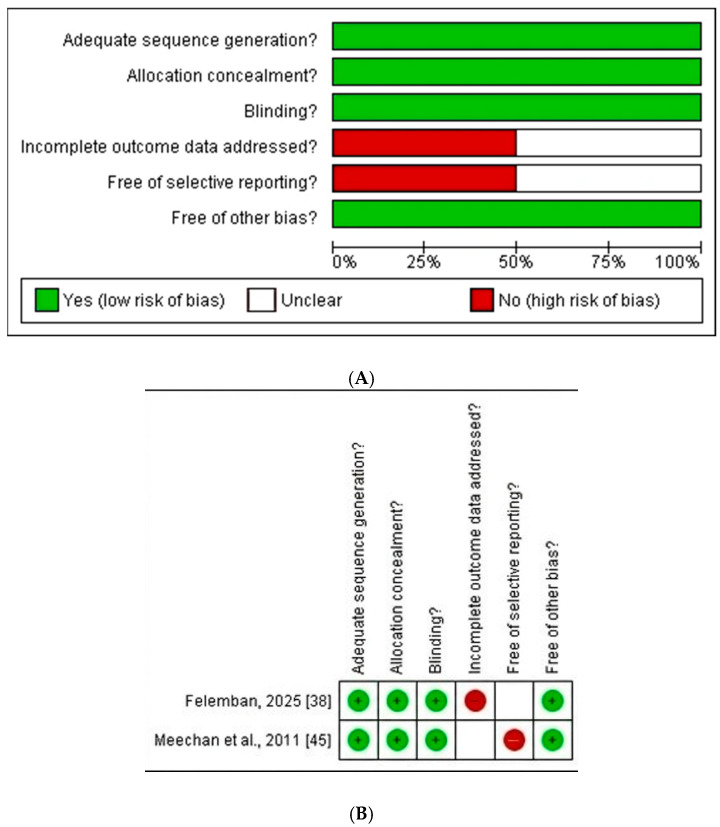
(**A**) Risk of bias graph and (**B**) Risk of bias summary [38,45].

The risk of bias assessment of the 8 included studies was performed using the Cochrane Risk Bias tool, and the results are in Figure 3A,B.

### 5.4. Quality Assessment of Included Studies

Most of the included studies were of high quality, while the remaining studies were of moderate quality, showing the authenticity and reliability of the included studies in this systematic review and meta-analysis shown in Table 3.

### 5.5. Primary Outcomes

#### 5.5.1. Pharmacology Knowledge

**Figure 4:**
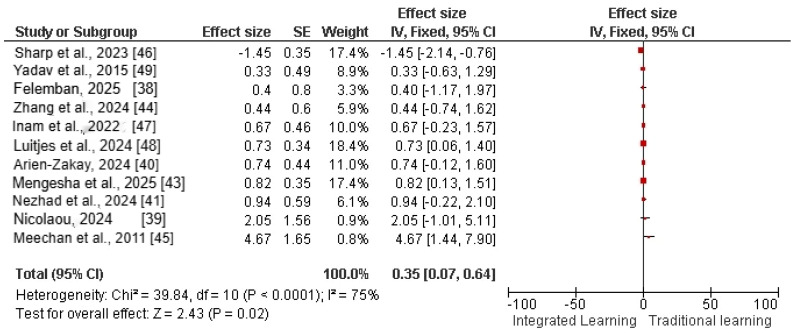
Forest plot of effect size of pharmacology knowledge among integrated learning vs. traditional learning [38,39,40,41,43,44,45,46,47,48,49]. The red squares represent the individual study effect estimates in the meta-analysis, positioned according to the magnitude and direction of each study’s effect. The size of each square reflects the study’s statistical weight, with larger squares indicating greater contribution to the pooled estimate, and the horizontal lines showing the 95 % CI.

**Figure 5:**
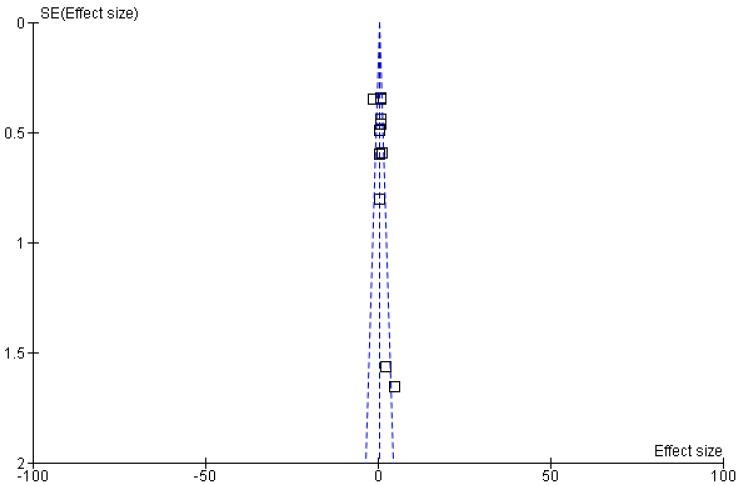
Funnel plot of effect size of pharmacology knowledge among integrated learning vs. traditional learning. The black squares represent the effect estimates from individual studies plotted against their standard errors. The blue dashed vertical line indicates the pooled effect estimate from the meta-analysis, while the sloping blue dashed lines represent the 95 % CI limits around the pooled effect, forming the funnel shape used to assess small study effects and potential publication bias.

Pharmacology knowledge outcomes were reported by all 11 included studies and were therefore eligible for quantitative synthesis. All included studies reported the pharmacology knowledge (assessed by final exam, professional pharmacology exams) as a major learning outcome of pharmacological curricula among the integrated learning group in comparison to the traditional learning (e.g., lecture-based learning) group. The pooled analysis was calculated by using a fixed-effects model and showed that pharmacology knowledge significantly improved among the integrated learning group in comparison to the traditional learning group [Effect size = 0.35 (95% CI: 0.07–0.64)], as shown in Figure 4. The test for heterogeneity revealed a Chi^2^ value of 39.84 (df = 10, *p* < 0.0001) and an *I*^2^ statistic of 75%, indicating substantial heterogeneity among the included studies. The moderate to low publication bias was reported among included studies, as predicted by funnel plots in Figure 5.

#### 5.5.2. Student Satisfaction

**Figure 6:**
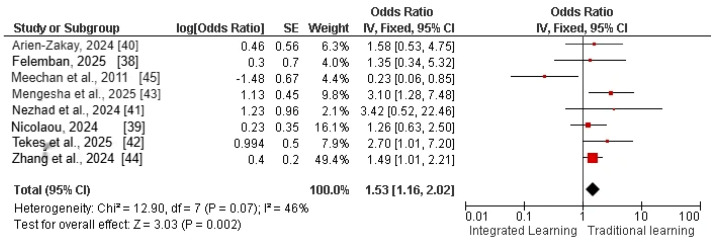
Forest plot of effect size of student satisfaction among integrated learning vs. traditional learning [38,39,40,41,42,43,44,45]. The red squares represent the individual study odds ratio estimates, positioned according to the magnitude and direction of each study’s effect. The size of each red square reflects the statistical weight of the study in the meta-analysis, with larger squares indicating greater influence on the pooled estimate. The horizontal lines through the squares show the 95 % CIs, and the black diamond at the bottom represents the pooled odds ratio with its corresponding 95 % CI.

Among 11 included studies, 8 studies reported student satisfaction (assessed by the Likert scale) as a major outcome of pharmacological curricula among integrated learning groups in comparison to traditional learning (e.g., lecture-based learning) groups. The pooled analysis was calculated by using a fixed-effects model and showed that student satisfaction levels significantly improved among the integrated learning group in comparison to the traditional learning group [odds ratio = 1.53 (95% CI: 1.16–2.02)], as shown in Figure 6. The test for heterogeneity revealed a Chi^2^ value of 12.90 (df = 7, *p* < 0.0001) and an *I*^2^ statistic of 46%, indicating moderate heterogeneity among the included studies.

## 6. Discussion

International pharmacy education aims to prepare students with the knowledge and skills for clinical or healthcare settings. The evolution of pharmacological curricula has been aiding the undergraduates and graduates of the medical, nursing, and dentistry fields to become qualified professionals and highly competent in their future careers [50]. The adoption of modern approaches such as problem-based, case-based, and hybrid models has promoted student-centred learning, strong prescribing behaviour, and therapeutic scenarios dealing with ultimate patient care in healthcare settings. Although several developing countries are relying on traditional pedagogical approaches in training medical students for future pharmacological practices, that cause hurdles in their global clinical interactions [11]. By keeping these challenges in view, this study aimed to evaluate and compare the learning outcomes of different pharmacological curricula across the globe, including Australia, by adopting a systematic review and meta-analysis research approach. The pooled analyses were meant to identify any general patterns of education practices as opposed to establishing a one-to-one correlation between countries whose cultures are different from each other, whose educational system is different from each other, and/or have different levels of resources available for education.

In this systematic review and meta-analysis, 11 studies published from 2000 to 2025 covering five curricula (such as case-based, problem-based, integrated teaching, team-based learning, and active learning against traditional lecture method-based curricula) of international pharmacy education were included. Through pooled analysis of 11 studies and 4140 medical or nursing students, the results of the analyses showed that students who were educated using an integrated/active learning curriculum type performed better on their examinations and had greater satisfaction with the education they received compared to students who were educated using a traditional lecture style as compared to traditional lecture-based curriculum. The heterogeneity ranged from 46% to 75%, which showed moderate to high variations among included studies due to factors such as variation in methodology, participant characteristics, or intervention protocols. Furthermore, the funnel plots showed low to moderate publication bias among included studies. Two randomised controlled trials [38,45] were evaluated as low-risk studies by the Cochrane Library tool. While the other 9 studies were mixed-design studies [39,42,48], quasi-experimental studies [40,41,49] and non-randomised studies (cross-sectional or observational studies) [43,46] that were assessed as moderate to high quality by the JBI checklist.

The findings of this study were consistent with previous studies that reported the effectiveness of modern integrated pharmacy curricula (such as problem-based, hybrid, and tech-based learning models) as compared to traditional learning approaches across the globe. For instance, Aref et al. [51] and Gill et al. [52] reported that the international trend of adopting integrated pharmacological curricula has improved pharmacology knowledge and students’ satisfaction levels among medical or nursing students. Furthermore, these studies supported modern pedagogical approaches in pharmacy education to enhance disease-oriented and clinical prescribing confidence among new medical professionals as well. According to Matinho et al. [53], many other authors of peer-reviewed education articles about the health professions failed to define the term ‘integrated’, explain how they linked their work to a pertinent theoretical framework, or describe how they actually pursued integrated learning. These failures represent lost opportunities in terms of best practice standards and make it more difficult for others to replicate and expand their research.

Pharmacology curricula in pharmacy and health-science education vary greatly across the globe in terms of their depth, structure, and pedagogical approach. However, Australian pharmacy education is notable for its close ties to international competency frameworks, clinical practice, and practical training. Although traditional lecture methods still dominated (approximately 90% of courses), pharmacology instruction varied across disciplines, and pharmacology lecture themes were discussed most frequently in medicine and with the most hours of involvement in pharmacy courses, according to a survey of Australian science, pharmacy, medicine, and nursing degree programmes [22]. However, various worldwide curricula emphasise a change towards competency-driven, outcome-based approaches as well as increased use of interprofessional education, simulation, and active learning. However, early experience placements, patient-centred pharmacy practice, and laboratory compounding training are all important components of the Australian pharmacy degree; according to one review, all 16 Bachelor of Pharmacy schools in Australia have specific compounding sections [54]. While 82% of Australian pharmacy programmes included pharmacogenomic/genetics content, the majority of learning objectives were still at the “Understand” level of Bloom’s Taxonomy rather than the “Apply” or “Evaluate” level. This indicates a gap with some international curricula that strive for higher-level competencies [55]. Furthermore, there are notable international parallels in graduate outcomes when the learning-outcome frameworks for pharmacies in Australia, Canada, the UK, and the US are aligned with the International Pharmaceutical Federation Global Competency Framework [56]. However, there are national differences in practice roles and teaching emphasis. In conclusion, Australian pharmacy education is clinically focused and competitive worldwide, but there is still room to improve the integration of higher-order competency training and applied pharmacology to match changing international requirements.

**Figure 7:**
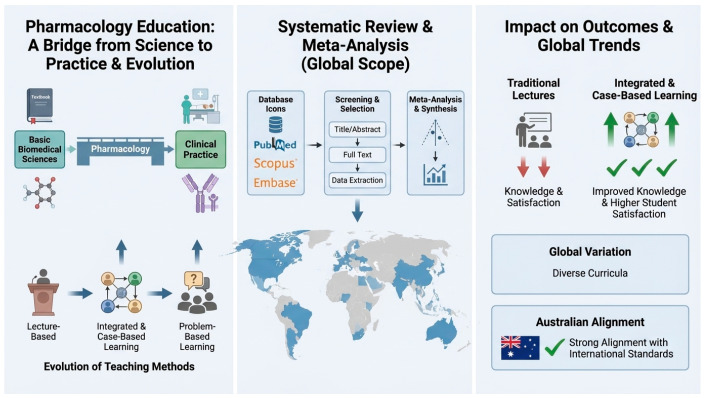
The international evolution of pharmacology education, systematic review methodology, and the impact of integrated teaching approaches on learning outcomes and international curriculum alignment. The progression from basic sciences to clinical practice, the workflow of evidence synthesis with global study representation, and the comparative impact of traditional versus integrated and case-based learning on student outcomes and curriculum alignment.

The present systematic review and meta-analysis provide a comprehensive synthesis of international pharmacological curricula spanning multiple pedagogical models and continents, including Australia, Asia, the Middle East, and Europe. Including a variety of study approaches, including observational, mixed-methods, quasi-experimental, and randomised controlled trials, allows for a comprehensive comparison of teaching efficacy across disciplines, which is one of this review’s main strengths. High analytical robustness was guaranteed by the exacting methodology, quality evaluation utilising Cochrane and JBI techniques, and adherence to PRISMA recommendations. The evaluation further improves the statistical power and generalisability of results by quantitatively combining data from 11 studies that included more than 4000 students. The comparative understanding of Australian pharmacy education, which gives curriculum designers more contextual depth and policy significance, is another noteworthy quality. Additionally, this study highlighted the influence of integrated and active learning models on student knowledge and satisfaction globally, supporting alignment between educational theory and applied pharmacology teaching practices (Figure 7).

## 7. Limitations

Despite its comprehensive approach, this review has several limitations. The included studies showed moderate to high heterogeneity (*I*^2^ = 46–75%), which probably reflected differences in evaluation instruments, instructional time, institutional settings, and participant demographics. Self-reported satisfaction measures were utilised in several studies, which may have introduced response bias and limited the ability to objectively analyse learning effects. Furthermore, it is more difficult to determine a causal relationship between curriculum style and educational success due to the prevalence of cross-sectional and quasi-experimental methods. The asymmetry of the funnel plot also revealed publication bias, indicating selective reporting of positive results. The underrepresentation of data from low-income nations is another drawback, which limits the findings’ applicability in educational situations with limited resources. Furthermore, direct comparison is made difficult by the lack of standardised outcome measurements between trials and the variation in the depth of pharmacological material. Future research should emphasise longitudinal designs, uniform evaluation standards, and inclusion of more diverse international institutions for a balanced perspective.

## 8. Conclusions

Overall, the findings reported that changing from traditional lecture-based pharmacology teaching to integrated, case-based, and active learning models significantly improves students’ pharmacological knowledge and satisfaction globally. These modern pedagogical approaches enhance stronger clinical reasoning, prescribing confidence, and patient-centred therapeutic understanding. The Australian pharmacy curriculum demonstrated a high degree of clinical integration and competency alignment with international frameworks, but still relies heavily on didactic methods in some areas. Bridging this gap requires further incorporation of applied pharmacology, interprofessional learning, and higher-order cognitive outcomes as emphasised in Bloom’s Taxonomy. Overall, the findings advocate for policy reforms and cross-national collaboration to strengthen pharmacology education and elevate professional standards in clinical practice worldwide.

## Figures and Tables

**Table 1:** PICOS framework with components and their description, used in this study.

Component	Description
Population (P)	Students of medicine, pharmacy, nursing, dentistry, and Allied health departments studying pharmacological curricula across global institutions.
Intervention (I)	Teaching approaches in pharmacology and clinical pharmacology, including integrated, PBL, flipped, simulation, VR, gamification, concept-based curricula, and competency frameworks.
Comparison (C)	Traditional Lecture-Based Teaching, Conventional Teaching Methods, Standard Curriculum.
Outcomes (O)	Primary outcomes are knowledge, Academic Performance, Knowledge Assessment, Clinical Competence, Objective Structured Clinical Examination, Prescribing Competency, Student Satisfaction scores.
Study Design (S)	Randomised Controlled Trial, Quasi-Experimental Study, Cross-Sectional Study, Mixed Methods Study, Curriculum Evaluation, national surveys, Delphi frameworks, and mixed methods.

**Table 2:** Characteristics of included studies of this systematic review and meta-analysis [38,39,40,41,42,43,44,45,46,47,48,49].

Author, Year	Region/Country	Study Population	Study Groups	Study Design	Assessment Tool	Institute	Study Period	Pharmacology Knowledge	Student Satisfaction (Likert Scale)
Felemban et al., 2025 [38]	Saudi Arabia	76 medical students	Case based learning = 40 Lecture based learning = 36	Randomised experimental study	General electronic survey	College of Medicine (COM) of King Saud bin Abdulaziz University for Health Sciences (KSAU-HS)	4 weeks 2023–2024	G1 = 4.08 (1.63) G2 = 4.75 (1.72) 0.40 [95%CI; 0.20–1.09]	G1 = 4.53 (0.74) G2 = 4.73 (0.51) 0.30 [95% CI; 0.10–0.80]
Nicolaou et al., 2024 [39]	United Kingdom	296 medical students	Case based learning = 58 Lecture based learning = 209	Mixed methods study	Student questionnaire	St. George’s University of London and University of Nicosia	2022–2024	G1 = 18.0 ± 5.02 G2 = 28.90 ± 5.39 2.05 [95%CI; 1.65–3.07]	G1 = 3.6 ± 0.8 G2 = 3.8 ± 0.9 0.23 [95% CI; 0.05–0.40]
Arien-Zakay et al., 2024 [40]	Israel	1275 Undergraduate nursing students (pre-clinical pharmacology)	Lecture-based learning (LBL) = 849 Blended learning = 426	Quasi-experimental cohort	Final in-class exam & end-of-course satisfaction course	Hebrew University of Jerusalem	2016–2020	G1 = 64.6 ± 16.0 G2 = 76.4 ± 16.0 0.74 = [95% CI; 0.56–1.1]	G1 = 328 7.8 ± 1.3 G2 = 133 8.3 ± 0.7 0.46 [95% CI; 0.34–0.95]
Nezhad et al., 2024 [41]	Middle East: Iran	240 undergraduate pharmacy students	Integrated group = 120 Traditional group = 120	Quasi-experimental, pre–post comparative study	Final exam & validated 5-point Likert-scale questionnaire	Tehran University of Medical Sciences	2021–2023	G1 = 82.4 ± 7.6 G2 = 74.8 ± 8.5 0.94	G1 = 4.3 ± 0.6 G2 = 3.5 ± 0.7 SMD = 1.23
Tekeş et al., 2025 [42] *	Turkey	97 medical students	Pre-test vs. post-test	Mixed-methods quantitative study	Likert scale	Çanakkale Onsekiz Mart University	2023–2024		G1 = 41.15 ± 4.11 G2 = 37.24 ± 3.73 0.994
Mengesha et al., 2025 [43]	Ethiopia	420 undergraduate health science students	Active learning = 210 Passive learning = 210	Cross-sectional, descriptive-analytical	Pharmacology knowledge & student satisfaction Likert scale	University of Gondar, College of Medicine and Health Sciences	October–December 2024	G1 = 14.6 ± 2.8 G2 = 12.1 ± 3.2 0.82 (0.61–1.04, *p* < 0.001)	G1 = 4.1 ± 0.6 G2 = 3.3 ± 0.8 1.13 (0.92–1.34, *p* < 0.01)
Zhang et al., 2024 [44]	China	245 undergraduate medical students	Integrated teaching = 119 Conventional teaching = 121	Prospective cohort study	Pharmacology drug exam	Anhui Medical University, Hefei, China	2019–2020	G1: 64 ± 17.6 G2: 56 ± 18.9 0.44 (0.14–0.74)	G1 = 22 G2 = 12 0.40
Meechan et al., 2011 [45]	UK	240 Undergraduate adult nursing students	Integrated teaching = 120 Conventional teaching = 120	Randomised Controlled Trial	Pharmacology Assessment Tool, & Self-Assessment Rating Score (SARS)—7-item, 4-point Likert	Institute of Health & Society, University of Worcester	2007–2008	G1 = 63.98 ± 2.91 G2 = 48.62 ± 3.59 4.67 [95% CI; 3.98, 5.36]	G1 = 2.33 (0.729) G2 = 3.57 (0.927) −1.48 = 95% CI ≈ [−1.88, −1.08]
Sharp et al., 2023 [46]	India	175 MBBS students	Modern curriculum = 98 Old curriculum = 77	Cross-sectional comparative study	Performance in exams	Govt. Medical College, Jalgaon, Maharashtra, India	2017–2019	G1 = 66.2 ± 7.3 G2 = 75.8 ± 5.4 −1.45 [95%CI: −0.45, −3.67]	
Inam et al., 2022 [47]	Pakistan	305 undergraduate MBBS students	Integrated curriculum = 137 Traditional curriculum = 168	Observational, cross-sectional study	Final Professional Pharmacology Examination	Azra Naheed Medical College, Superior University	April–May 2022	G1 = 188.04 ± 31.8 G2 = 204.98 ± 17.0 0.67 (0.44–0.90)	
Luitjes et al., 2024 [48]	Netherlands	686 students	Team-based learning (TBL) = 196 Conventional = 490	Mixed-method study	Final exam and feedback questionnaire	Utrecht University Medical School, UMC Utrecht	2021–2023	G1 = 76.74 ± 14.85 G2 = 64.33 ± 17.85 0.73 (0.56–0.90)	
Yadav et al., 2015 [49]	India	330 undergraduate medical students	Integrated teaching = 165 Conventional = 165	Quasi-experimental design	Pre and post knowledge scores	Government Medical College & New Civil Hospital, Surat	2013–2014	G1 = 24.48 ± 6.58 G2 = 19.52 ± 4.87 G = 0.33 (0.08–0.57)	

Notes: G1 = GROUP 1, G2 = GROUP 2. * Although twelve studies were initially identified as eligible, one study was excluded from the qualitative synthesis and meta-analysis due to insufficient extractable quantitative outcome data. Consequently, eleven studies were included in the final analysis and quality assessment.

**Table 3:** Quality Assessment of Included studies by JBI checklist.

Author (Year)	Q1 Similar Groups	Q2 Exposure Measured	Q3 Valid Exposure	Q4 Confounders Identified	Q5 Confounders Addressed	Q6 Outcome Free at Start	Q7 Valid Outcomes	Q8 Follow-Up Sufficient	Q9 Complete Follow-Up	Q10 Incomplete Follow-Up Addressed	Q11 Stats Appropriate	Total (Out of 11)	Quality
Nicolaou et al., 2024 [39]	✓	✓	✓	?	✗	✓	✓	✓	✗	✗	✓	7/11	Moderate–High
Arien-Zakay et al., 2024 [40]	✓	✓	✓	?	✗	✓	✓	✓	✓	?	✓	8/11	High
Nezhad et al., 2024 [41]	✓	✓	✓	✓	?	✓	✓	✓	✓	?	✓	9/11	High
Tekeş et al., 2025 [42] *	✓	✓	✓	?	✗	✓	✓	✓	✗	✗	✓	7/11	Moderate–High
Mengesha et al., 2025 [43]	✓	✓	✓	✓	✓	✓	✓	✓	?	?	✓	9/11	High
Zhang et al., 2024 [44]	✓	✓	✓	✗	✗	✓	✓	✓	?	✗	✓	7/11	Moderate–High
Meechan et al., 2011 [45]	✓	✓	✓	?	✗	✓	✓	✓	✗	✗	✓	7/11	Moderate–High
Sharp et al., 2023 [46]	✓	✓	✓	?	✗	✓	✓	✓	✓	✗	✓	8/11	High
Inam et al., 2022 [47]	✓	✓	✓	?	✗	✓	✓	✓	✓	✗	✓	8/11	High
Luitjes et al., 2024 [48]	✓	✓	✓	✗	✗	✓	✓	✓	?	✗	✓	7/11	Moderate–High
Yadav et al., 2015 [49]	✓	✓	✓	?	✗	✓	✓	✓	✗	✗	✓	7/11	Moderate–High

Notes: ✓ = Yes, ✗ = No, ? = Not clear. * Although twelve studies were initially identified as eligible, one study was excluded from the qualitative synthesis and meta-analysis due to insufficient extractable quantitative outcome data. Consequently, eleven studies were included in the final analysis and quality assessment.

## Data Availability

No new data were created or analysed in this study.

## Supplementary Materials

The following supporting information can be downloaded at: https://www.mdpi.com/article/10.3390/pharmacy14010027/s1, PRISMA checklist.
