# Supplementary material for: International Benchmarking of Pharmacology Curricula and Prescribing Related Learning Outcomes, Implications for Australian Health Professional Education: A Systematic Review and Meta-Analysis
Source: Pharmacy (Basel). 2026 Feb 3;14(1):27. doi: 10.3390/pharmacy14010027 (PMC12922130; doi:10.3390/pharmacy14010027)
Supplement: Supplementary file 1 [file pharmacy-14-00027-s001.zip › pharmacy-4103187-supplementary.pdf]

PRISMA 2020 Checklist

Section and Topic	Item #	Checklist item	Location where item is reported
**TITLE**			
Title	1	Identify the report as a systematic review.	Title page and running title. The title explicitly states “A Systematic Review and Meta-analysis”.
**ABSTRACT**			
Abstract	2	See the PRISMA 2020 for Abstracts checklist.	Abstract. Background, Methods, Results, and Conclusion are clearly structured and include databases searched, registration number, number of included studies, and pooled outcomes.
**INTRODUCTION**			
Rationale	3	Describe the rationale for the review in the context of existing knowledge.	Introduction, paragraphs 1 to 6. The manuscript describes global variation in pharmacology curricula, gaps in standardisation, and the need for benchmarking, including Australian context.
Objectives	4	Provide an explicit statement of the objective(s) or question(s) the review addresses.	Section 2 Objectives and Section 3 Key Questions. Explicit objectives and research questions are clearly stated.
**METHODS**			
Eligibility criteria	5	Specify the inclusion and exclusion criteria for the review and how studies were grouped for the syntheses.	Section 4.2 Eligibility Criteria, including subsections 4.2.1 Inclusion Criteria and 4.2.2 Exclusion Criteria.
Information sources	6	Specify all databases, registers, websites, organisations, reference lists and other sources searched or consulted to identify studies. Specify the date when each source was last searched or consulted.	Section 4.3 Search Strategy. Databases listed include MEDLINE (PubMed), EMBASE, CINAHL, PsycINFO, and the Cochrane Library. Date range specified as January 2000 to October 2025.
Search strategy	7	Present the full search strategies for all databases, registers and websites, including any filters and limits used.	Section 4.3 Search Strategy. Full Boolean search strategy for PubMed is provided, with statement that similar strategies were applied to other databases.
Selection process	8	Specify the methods used to decide whether a study met the inclusion criteria of the review, including how many reviewers screened each record and each report retrieved, whether they worked independently, and if applicable, details of automation tools used in the process.	Section 4.4 Study Selection and Data Extraction. Two reviewers independently screened titles, abstracts, and full texts. Disagreements resolved by discussion or third reviewer.
Data collection process	9	Specify the methods used to collect data from reports, including how many reviewers collected data from each report, whether they worked independently, any processes for obtaining or confirming data from study investigators, and if applicable, details of automation tools used in the process.	Section 4.4 Study Selection and Data Extraction. Data extracted independently by reviewers. Extracted items are clearly listed.
Data items	10a	List and define all outcomes for which data were sought. Specify whether all results that were compatible with each outcome domain in each study were sought (e.g. for all measures, time points, analyses), and if not, the methods used to decide which results to collect.	Section 4.1 PICOS Framework and Section 4.4 Data Extraction. Outcomes include pharmacology knowledge, prescribing competence, OSCE performance, and student satisfaction.
	10b	List and define all other variables for which data were sought (e.g. participant and intervention characteristics, funding sources). Describe any assumptions made about any missing or unclear information.	Section 4.4 Data Extraction. Variables include study design, population, curriculum type, comparator, assessment tools, country, and study period.
Study risk of bias assessment	11	Specify the methods used to assess risk of bias in the included studies, including details of the tool(s) used, how many reviewers assessed each study and whether they worked independently, and if applicable, details of automation tools used in the process.	Section 4.6 Risk of Bias Assessment and Figure 3. Cochrane Risk of Bias tool used for RCTs. JBI checklist used for non-randomised studies.

PRISMA 2020 Checklist

Section and Topic	Item #	Checklist item	Location where item is reported
Effect measures	12	Specify for each outcome the effect measure(s) (e.g. risk ratio, mean difference) used in the synthesis or presentation of results.	Section 4.7 Statistical Analysis. Odds ratios with 95 percent confidence intervals for dichotomous outcomes and standardised mean differences for continuous outcomes.
Synthesis methods	13a	Describe the processes used to decide which studies were eligible for each synthesis (e.g. tabulating the study intervention characteristics and comparing against the planned groups for each synthesis (item #5)).	Section 5 Results and Section 5.5 Primary Outcomes. Studies eligible for meta-analysis were those reporting comparable quantitative outcomes.
	13b	Describe any methods required to prepare the data for presentation or synthesis, such as handling of missing summary statistics, or data conversions.	Section 4.7 Statistical Analysis. Use of Hedges g, handling of continuous and dichotomous data, and model selection based on heterogeneity described.
	13c	Describe any methods used to tabulate or visually display results of individual studies and syntheses.	Tables 1 and 2, Figures 2 to 7. Forest plots, funnel plots, and tabulated study characteristics provided.
	13d	Describe any methods used to synthesize results and provide a rationale for the choice(s). If meta-analysis was performed, describe the model(s), method(s) to identify the presence and extent of statistical heterogeneity, and software package(s) used.	Section 4.7 Statistical Analysis. Fixed and random effects models described. Heterogeneity assessed using Chi-squared test and I^2 statistic. RevMan 5.4 software used.
	13e	Describe any methods used to explore possible causes of heterogeneity among study results (e.g. subgroup analysis, meta-regression).	Section 5.5 Primary Outcomes. Heterogeneity quantified using I^2. Sources discussed narratively in Discussion.
	13f	Describe any sensitivity analyses conducted to assess robustness of the synthesized results.	Not performed. This is not explicitly reported. This should be stated as “not conducted” if submitting the checklist to the journal.
Reporting bias assessment	14	Describe any methods used to assess risk of bias due to missing results in a synthesis (arising from reporting biases).	Section 5.5 Primary Outcomes and Figure 5 Funnel Plot. Publication bias assessed visually using funnel plots.
Certainty assessment	15	Describe any methods used to assess certainty (or confidence) in the body of evidence for an outcome.	Not formally assessed using GRADE. This is not reported and should be marked as “not assessed”.
**RESULTS**			
Study selection	16a	Describe the results of the search and selection process, from the number of records identified in the search to the number of studies included in the review, ideally using a flow diagram.	Section 5.1 Study Selection and Figure 2 PRISMA Flow Diagram. Numbers at each stage are clearly reported.
	16b	Cite studies that might appear to meet the inclusion criteria, but which were excluded, and explain why they were excluded.	Figure 2 PRISMA Flow Diagram and Section 5.1. Reasons for exclusion are summarised.
Study characteristics	17	Cite each included study and present its characteristics.	Section 5.2 Characteristics of Included Studies and Table 1.
Risk of bias in studies	18	Present assessments of risk of bias for each included study.	Section 5.3 Risk of Bias Assessment and Figure 3.
Results of individual studies	19	For all outcomes, present, for each study: (a) summary statistics for each group (where appropriate) and (b) an effect estimate and its precision (e.g. confidence/credible interval), ideally using structured tables or plots.	Table 1 and forest plots in Figures 4 and 6. Effect sizes and confidence intervals are presented.
Results of syntheses	20a	For each synthesis, briefly summarise the characteristics and risk of bias among contributing studies.	Section 5.5 Primary Outcomes. Characteristics and quality of contributing studies are summarised.
	20b	Present results of all statistical syntheses conducted. If meta-analysis was done, present for each the summary estimate and its precision (e.g.	Figures 4 and 6, with pooled effect estimates, confidence intervals, and heterogeneity statistics.

PRISMA 2020 Checklist

Section and Topic	Item #	Checklist item	Location where item is reported
		confidence/credible interval) and measures of statistical heterogeneity. If comparing groups, describe the direction of the effect.	
	20c	Present results of all investigations of possible causes of heterogeneity among study results.	Section 5.5 and Discussion. Heterogeneity discussed narratively.
	20d	Present results of all sensitivity analyses conducted to assess the robustness of the synthesized results.	Not conducted. This should be explicitly stated as “not performed”.
Reporting biases	21	Present assessments of risk of bias due to missing results (arising from reporting biases) for each synthesis assessed.	Figure 5 Funnel Plot and Section 5.5.
Certainty of evidence	22	Present assessments of certainty (or confidence) in the body of evidence for each outcome assessed.	Not formally assessed. This should be marked as “not assessed”.
**DISCUSSION**			
Discussion	23a	Provide a general interpretation of the results in the context of other evidence.	Section 6 Discussion. Results interpreted in relation to global literature and prior studies.
	23b	Discuss any limitations of the evidence included in the review.	Section 7 Limitations.
	23c	Discuss any limitations of the review processes used.	Section 7 Limitations. Review design and heterogeneity are discussed.
	23d	Discuss implications of the results for practice, policy, and future research.	Section 6 Discussion and Section 8 Conclusion. Implications for curriculum reform, policy, and future research are discussed.
**OTHER INFORMATION**			
Registration and protocol	24a	Provide registration information for the review, including register name and registration number, or state that the review was not registered.	Abstract and Section 4 Methods. PROSPERO registration number CRD420251207753.
	24b	Indicate where the review protocol can be accessed, or state that a protocol was not prepared.	Methods. Protocol registered in PROSPERO. No separate protocol publication stated.
	24c	Describe and explain any amendments to information provided at registration or in the protocol.	Not applicable. No amendments reported.
Support	25	Describe sources of financial or non-financial support for the review, and the role of the funders or sponsors in the review.	Funding section. No external funding declared.
Competing interests	26	Declare any competing interests of review authors.	Conflicts of Interest section. No competing interests declared.
Availability of data, code and other materials	27	Report which of the following are publicly available and where they can be found: template data collection forms; data extracted from included studies; data used for all analyses; analytic code; any other materials used in the review.	Not explicitly stated. Data derived from published studies. This can be marked as “data available upon reasonable request” if required by the journal.

From: Page MJ, McKenzie JE, Bossuyt PM, Boutron I, Hoffmann TC, Mulrow CD, et al. The PRISMA 2020 statement: an updated guideline for reporting systematic reviews. BMJ 2021;372:n71. doi: 10.1136/bmj.n71. This work is licensed under CC BY 4.0. To view a copy of this license, visit https://creativecommons.org/licenses/by/4.0/
